# Regional variations in mortality and causes of death in Israel, 2009–2013

**DOI:** 10.1186/s13584-017-0164-1

**Published:** 2017-08-01

**Authors:** Ethel-Sherry Gordon, Ziona Haklai, Jill Meron, Miriam Aburbeh, Inbal Weiss Salz, Yael Applbaum, Nehama F. Goldberger

**Affiliations:** Division of Health Information, Ministry of Health, Yirmiyahu, 39, 9446724 Jerusalem, Israel

**Keywords:** SMR, Regional differences, Causes of death, Periphery, Medical services

## Abstract

**Background:**

Regional variations in mortality can be used to study and assess differences in disease prevalence and factors leading to disease and mortality from different causes. To enable this comparison, it is important to standardize the mortality data to adjust for the effects of regional population differences in age, nationality and country of origin.

**Methods:**

Standardized mortality ratios (SMR) were calculated for the districts and sub-districts in Israel, for total mortality by gender as well as for leading causes of death and selected specific causes. Correlations were assessed between these SMRs, regional disease risk factors and socio-economic characteristics. Implications for health policy were then examined.

**Results:**

Total mortality in the Northern District of Israel was not significantly different from the national average; but the Haifa, Tel Aviv, and Southern districts were significantly higher and the Jerusalem, Central, Judea and Samaria districts were lower.

Cancer SMR was significantly lower in Jerusalem and not significantly higher in any region. Heart disease and diabetes SMRs were significantly higher in many sub-districts in the north of the country and lower in the south. SMRs for septicemia, influenza/pneumonia, and for cerebrovascular disease were higher in the south. Septicemia was also significantly higher in Tel Aviv and lower in the North, Haifa and Jerusalem districts. SMRs for accidents, particularly for motor vehicle accidents were significantly higher in the peripheral Zefat and Be’er Sheva sub-districts.

**Conclusion:**

The SMR, adjusted for age and ethnicity, is a good method for identifying districts that differ significantly from the national average. Some of the regional differences may be attributed to differences in the completion of death certificates. This needs to be addressed by efforts to improve reporting of causes of death, by educating physicians.

The relatively low differences found after adjustment, show that factors associated with ethnicity may affect mortality more than regional factors. Recommendations include encouraging good eating habits, exercise, cancer screening, control of hypertension, reduction of smoking and improving road infrastructure and emergency care access in the periphery.

**Electronic supplementary material:**

The online version of this article (doi:10.1186/s13584-017-0164-1) contains supplementary material, which is available to authorized users.

## Background

Data on geographic mortality patterns by cause of death are an important, readily available indicator which may reflect disease prevalence and quality of treatment, and can guide policy makers on interventions and inequalities that need to be addressed.

Israel has a unique multi-ethnic population, the majority of whom are immigrants and their offspring, who came from all the continents of the world. In addition, about 20% of the population are Israeli Arabs. Ethnic differences in mortality are well documented, such as in the ‘Health in Israel’ publication of the Ministry of Health (MOH) [1]. For example, Arab mortality has been reported there as higher for most causes, in particular diabetes and heart disease, but was lower for some cancers, such as female breast cancer. Jews of European-American origin had higher rates of colorectal and breast cancer while males of Asian origin had high mortality from liver diseases. In order to correctly compare regional differences, it is therefore necessary to adjust for the regional differences in ethnic composition (country of origin and nationality). This was undertaken in the past in a series of papers presented by Ginsberg et al. [2–4] on standardized mortality ratios in Israel, for the years 1967–1978, 1983–1986, and 1987–1994. In the first and third papers, results were presented for Jews only, age and gender standardized in the first paper, and also for continent of birth in the third paper. The second paper presented, in addition, results for Jews and non-Jews standardized for age, gender, religion and continent of birth.

In the thirty years since Ginsberg et al.’s results, there have been major demographic and mortality profile changes in Israel. Israel absorbed almost 900,000 immigrants from the Former Soviet Union (FSU), the majority between 1990 and 1995, who comprised 10% of the population in 2012. About 60,000 more immigrants also came from Ethiopia, many in the big wave of immigration in 1990–1991. New cities were built, with mostly younger population, in the Ramla, Petah Tiqwa and Judea and Samaria sub-districts, such as Modi’in,Shoham, Modi’in Ilit and Beitar Illit. Mortality rates have decreased considerably and since the end of the 90’s the leading cause of death for both genders is now cancer, followed by heart disease, cerebrovascular disease and diabetes, as reported in the Ministry of Health’s publication, leading causes of death in Israel, 2000–2012 [5]. Mortality rates for cardiovascular diseases, in particular, have decreased sharply [5]. The proportion of Israeli born population has increased, including many of mixed ethnic origins.

The Central Bureau of Statistics (CBS) in Israel published age standardized mortality rates by region and gender for those aged 45 and over in 2005–2009 [6] and for all ages by causes of death, regions, gender and nationality for 2006–2008 [7], but not a regional measure standardizing for both age and ethnicity.

We have presented another measure on a regional level in Israel; mortality rates from causes amenable to health care, 2007–2009 [8]. However, also these rates were not adjusted for ethnicity. Therefore, although this measure showed considerable variation between regions, this may be due in part to differences in the ethnic composition of the regional population, which may mask other regional differences.

Geographic distribution of mortality has also been studied worldwide and used to assess and improve care and outcomes. Canada first published a mortality atlas in 1980 [9], followed by the USA in 1996 [10], the European Union [11] and Australia [12].

Mortality differences in Europe for the mainland regions of EU countries in the early 1990’s were discussed in a paper by Shaw et al. [13], and an updated discussion on cardiovascular mortality patterns in Europe in 2000 was presented by Muller-Nordhorn [14]. Filate et al. presented data in 1995–1997 on regional variations in cardiovascular mortality in Canada together with analyses to find relationships between the different rates and regional risk factors and characteristics [15]. Sepsis mortality variations in the USA in 1995–2005 were described by Wang et al. [16].

The mortality atlases show disease patterns and their possible causes. An important initial observation in the Eurostat atlas [11] is that sharp international boundaries between causes of death may be due to diagnostic and coding practices, although this is less of a problem for some specific diseases such as lung cancer and transport accidents, which are more likely to be coded correctly. Another problem noted there is of the deaths coded as ‘sudden death’ of unknown origin, which may often be cardiovascular, but can only be reliably identified by autopsy. Therefore countries with higher autopsy rates tend to have higher cardiovascular mortality. This atlas notes that higher diversity in mortality rates, such as those for cardiovascular disease, suggest a high potential for prevention by effective health policy. For example, ischemic heart disease has higher levels in the north and east of Europe, traditionally explained by the ‘Mediterranean paradox’, which suggests that a ‘Mediterranean’ diet rich in olive oil, legumes, unrefined cereals, and fruit and vegetables, and a moderate consumption of alcohol, can reduce heart disease, even with a relatively high animal fat consumption [11]. The importance of encouraging this type of healthy diet as well as lifestyle changes, such as increased physical activity and smoking cessation is indicated by this data.

Our objectives in this study in Israel were to determine whether there are currently significant regional differences in overall and cause specific mortality, adjusted for age and ethnicity, and to determine how these differences compare with those reported by Ginsberg et al. [3] about thirty years earlier. We then assessed the implications of these results on health policy. We present the Standardized Mortality Ratio (SMR), to compare regional mortality to the national average rates by cause, after adjusting for the effects of different distributions of age and ethnicity (Arabs, and Jews and Others by continent of birth).

Going into the study, we expected to find regional differences in the SMRs and assumed these would reflect factors such as variations in environmental conditions, socio-economic conditions, education, lifestyle, genetics, religiosity, access to healthcare, and comorbidity. As in the paper of Filate et al. [15], we investigated some of these relationships by calculating the correlations of SMRs for overall mortality and leading causes with selected socio-economic measures and risk factors.

## Methods

Israeli mortality data was taken from the nationwide database of causes of death prepared by the CBS for the years 2009–2013, using underlying cause of death coded according to ICD-10.

Population data was also supplied by the CBS for these years, by age group, gender, nationality and continent of birth, district and sub-district.

Causes of death were chosen from the “List of 113 causes of death” of the WHO, associated with ICD-10, with the addition of dementia, which has been increasing in Israel in recent years [5], and was recently added to the Eurostat database.

Israel is divided into 7 districts. 4 are divided into 13 sub-districts, while the remaining 3 districts of Jerusalem, Tel Aviv and Judea and Samaria are not divided. Hence there are 16 distinct geographical units, for which the SMR was calculated, in addition to that for the other 4 districts. A map showing the districts and sub-districts in included as Additional file 1. The divisions are for administrative purposes and do not, in general, reflect geographical differences.

The 16 distinct geographical units vary in population between the large Tel Aviv and Jerusalem districts with populations of 1,290,000 and 957,000 respectively, to the Golan, Kinneret and Zefat sub-districts with populations of 43,000, 105,000 and 109,000 respectively, in 2011 (shown in Table 1). A table of demographic, socio-economic, risk factor and health service characteristics of sub-districts is shown in Additional file 2. There are significant differences between regions in many of these characteristics. For example, the new immigrant population (immigrated since 1990) is highest in the Ashkelon sub-district (34%) followed by Haifa (25%) and Beer Sheva (22%) compared to the national average of 17%. The highest proportion of young people under 15 is found in Judea and Samaria (41%) followed by Jerusalem (35%) and Beer Sheva (34%) compared to the national average of 28%. The sub-district with the largest proportion of elderly, over 65, is Haifa (16%) followed by Tel Aviv (15%) compared to the national average of 10%.

**Table 1:**
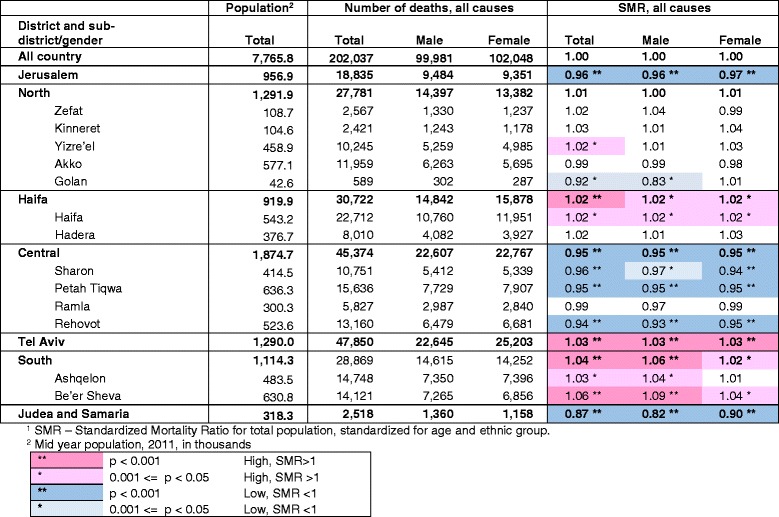
Numbers of deaths and SMR^1^ for total mortality by district, sub-district and gender, 2009–2013

Indirect standardization to calculate the SMR was used since it is preferred where some cells have small values, as were some of the denominator population cells in our data. The groups used for calculation were based on 6 age categories (under 25, 25–44, 45–54,55–64, 65–74, 75+), and 5 ethnic groups (Jews and Others divided by their continent of birth: Israeli, Europe-American, Asian and African, and Arabs). The 65–74 and 75 and over age groups were pooled for the Arabs due to small numbers in some sub-districts, giving 29 age-ethnic groups used for standardization.

For each age-ethnic group, the group specific overall mortality rate and cause specific rates were calculated for the years 2009–2013 for the total population of Israel, used as reference population. These rates were then applied to each district and sub-district according to their specific age-ethnic population distribution to obtain an expected number of deaths. The SMR was calculated as the ratio between the observed number of deaths and this expected number of deaths in the region:

$$ SMR=\frac{observed\  number\  of\  deaths}{expected\  number\  of\  deaths}=\frac{N}{\sum_i{p}_i{r}_i} $$

where N = observed number of deaths in district/sub-district.

*p*_*i*_ = population size of i^th^ age-ethnic group in district/subdistrict.

*r*_*i*_ = total national mortality rate for i^th^ age-ethnic group.

An SMR greater than 1 indicates that the number of actual deaths was higher than expected, based on average national rates, while an SMR less than 1 indicates a lower number of deaths than expected. Gender specific SMRs were similarly calculated using the total population rates by gender as reference.

We present the SMR for total mortality, leading causes in Israel [5] and for specific causes, by district and sub-district. Confidence intervals for the SMR were calculated by the method suggested by Ulm [17]. We marked values which were significant on the *p* < 0.001 and *p* < 0.05 level in the tables. This enables trends across the country to be seen, which may include results of lower significance. However we report and discuss only the highly significant results, given the large number of comparisons. It should be noted that the SMR reflects the comparison of each region separately with the national rates and therefore should not be compared between regions.

Pearson correlations were calculated between the total SMR, SMRs by gender and SMRs for leading causes with selected socio-economic measures, disease prevalence (cancer, diabetes and hypertension) and the risk factor of smoking, in 15 distinct regions. The Golan sub-district was excluded from this analysis, because of uncertainty in its characteristics and wide confidence intervals for SMR, due to its small size. The measures were taken from the health profile of districts published by the CBS and the Ministry of Health (MOH) [18], or other CBS survey data.

Although not all findings have an obvious explanation, we suggest some factors that may be the cause of geographical differences.

## Results

### Total mortality in Israel by district and sub-district, 2009–2013

**Fig. 1:**
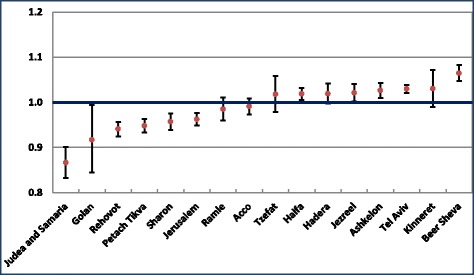
SMR by sub-district, 2009–2013. Standardized for age and ethnic group, with 95% CI error bars

There were about 202,000 deaths in Israel between 2009 and 2013. Figure 1 shows the SMR for total mortality by sub-district with 95% confidence intervals, and Table 1 shows the number of deaths and SMR values for total mortality by district, sub-district and gender. SMRs showed mortality significantly lower than the national average by 4–6% in the Rehovot, Petah Tiqwa, Sharon and Jerusalem sub-districts and 13% lower in Judea and Samaria. Significantly higher rates were found in the Be’er Sheva sub-district, and in Tel Aviv 6% and 3% higher than the national average respectively. In other sub-districts differences with the national average were smaller or not statistically significant. . The gender specific SMRs were generally similar to the total.

For comparison, we calculated these SMRs adjusting for age only and not ethnic origin. The range of SMR was greater, as shown in Additional file 3, between 18% lower than the national average in Judea and Samaria to 10% higher in Be’er Sheva, and there were also significant changes in some sub-districts compared to that adjusted for ethnic origin as well.

### Mortality for leading causes of death in Israel by district and sub-district, 2009–2013

Table 2 shows SMR values for mortality from leading causes of death in Israel by district and sub-district. In line with their low total mortality, the Jerusalem, Central and Judea and Samaria districts were found to have lower mortality than the national average for most leading causes Significantly low values were found in the Jerusalem district for septicemia (0.75), cancer (0.93), and cerebrovascular disease (0.90), in the Judea and Samaria district for diabetes (0.61), and in the Central district for septicemia (0.91), heart disease (0.94) and influenza/pneumonia (0.89). The only exceptions in Jerusalem were dementia, where the SMR was significantly high (SMR = 1.25) and heart disease (SMR = 1.06), statistically of lower significance.

**Table 2:**
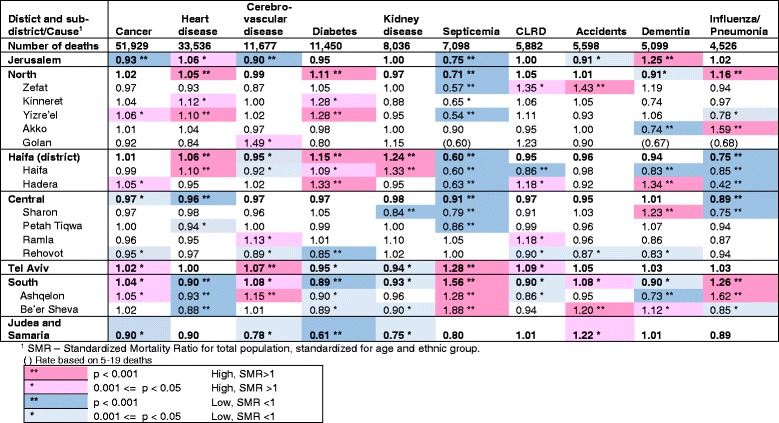
SMR^1^ for mortality by leading causes of death, district and sub-district, 2009–2013

^**2**^ **ICD10 codes:** Cancer (malignant neoplasms) C00-C97, Heart diseases I00-I09, I11,I13,I20-I51, Cerebrovascular diseases I60-I69, Diabetes mellitus E10-E14, Kidney diseases (nephritis, nephrotic syndrome and nephrosis) N00-N07, N17-N19, N25-N27, Septicemia A40-A41, CLRD (Chronic Lower Respiratory Disease) J40-J47 Accidents V01-X59, Y85-Y86, Dementia F01,F03, Influenza/pneumonia J09-J18

In the Northern and Haifa districts SMRs for septicemia were low compared to the national average, while those for diabetes and heart disease were high, in particular for diabetes in the Yizre’el and Hadera sub-districts and for heart disease in the Yizre’el and Haifa sub-districts. SMRs were also high for influenza/pneumonia in the Northern district, with a particularly high SMR in the Akko sub-district (SMR = 1.59), but were low in the Haifa and Hadera sub-districts (SMR = 0.85 and 0.42, respectively). In the Haifa sub-district, dementia’s SMR was also low (SMR = 0.83), but that of kidney disease was significantly high (SMR = 1.33). SMR for dementia was also significantly high in the Hadera sub-district (SMR = 1.34), but low in the Akko sub-district (SMR = 0.74). SMR for mortality due to accidents showed 43% higher rates than expected from national ones in the Zefat sub-district.

In line with their high total SMRs, Tel Aviv and the Southern district and sub-districts showed high SMRs for several leading causes. In the Tel Aviv district significantly high SMRs were found for cerebrovascular disease (1.07) and septicemia (1.28). Similarly in the Southern district, the SMR was high for septicemia (1.56) and also for influenza/pneumonia (1.26), in particular in the Ashquelon sub-district, where cerebrovascular disease SMR was also significantly high (SMR = 1.15). Accident SMR was significantly high in the Be’er Sheva sub-district (SMR = 1.20). However, the SMRs for heart disease and diabetes were significantly low in the Southern district (0.89 and 0.90, respectively).

### Mortality for selected sub-causes of death in Israel by district and sub-district, 2009–2013

Table 3 shows the SMR values for selected sub-groups of the leading causes of interest: the major cancer groups, ischemic heart disease, motor vehicle traffic accidents (the largest sub-group of accidents), and suicide. As for total cancer mortality, we found little regional variation for particular subgroups, with the exception of lung cancer mortality which was significantly lower in the Jerusalem district (SMR = 0.79) and higher in the Tel Aviv district (SMR = 1.08) and colorectal cancer mortality, higher in the Ashquelon sub-district (SMR = 1.17).

**Table 3:**
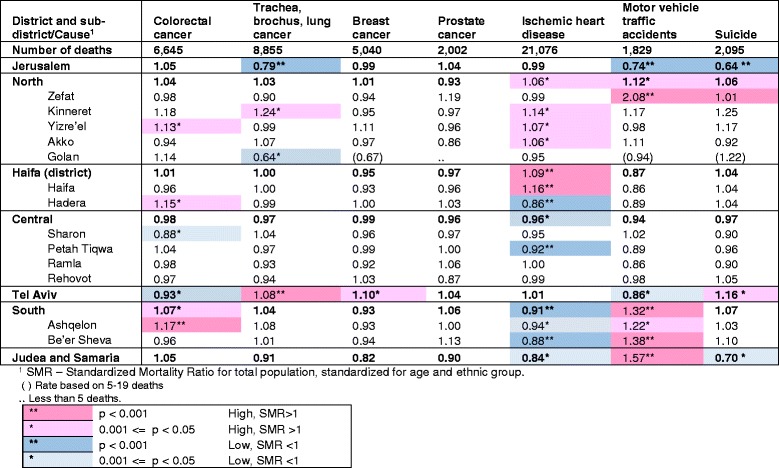
SMR^1^ for mortality by selected causes of death, by district and sub-district, 2009–2013

^**2**^ **ICD10 codes:** Colorectal cancer C18-C21,Trachea,bronchus,lung cancer C33-C34, Breast cancer C50, Prostate cancer C61, Ischemic heart disease I20-I25, Motor vehicle traffic accidents V02-V04,V09,V12-V14,V19-V79,V82-V87,V89, Suicide X60-X84, Y87.0

Ischemic heart disease mortality was significantly higher than the national average in the Haifa sub-district and lower in the Hadera, Petah Tiqwa and Be’er Sheva sub- districts.

Motor vehicle traffic accident deaths were particularly high in the Zefat and Be’er Sheva sub-districts (SMR = 2.08 and 1.38, respectively), and in the Judea and Samaria district (SMR = 1.57), and significantly low in the Jerusalem district (SMR = 0.74). Suicide was also significantly lower in the Jeusalem district (SMR = 0.64).

Additional files 4, 5 and 6 show the SMRs presented in Tables 1, 2 and 3 with 95% confidence intervals.

### Correlations of SMR with regional characteristics

Table 4 shows the Pearson correlation with regional socio-economic, disease prevalence and risk factor characteristics.

**Table 4:**
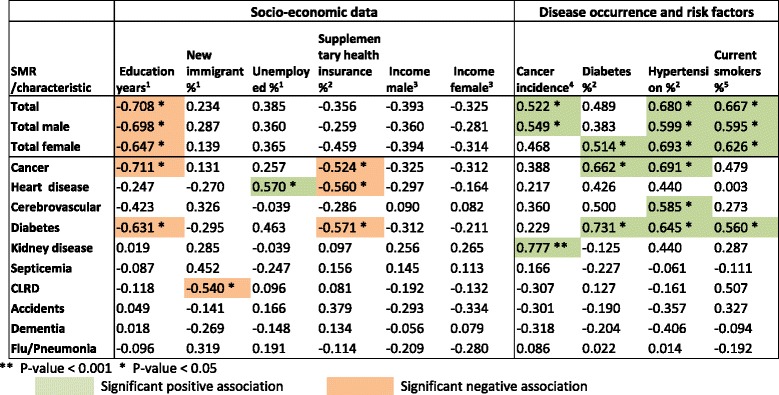
Pearson correlations of SMRs with socio-economic, disease and risk factors for 15 regions

Sources and definitions.

1 Calculated from CBS Labour force survey data, average for 2009–2011 for population aged 15 and over. Immigrants - from 1990. Unemployed: for last year.

2 CBS and MOH Health and social profile of localities, 2005–2009 [18], based on Health Survey, 2009, for ages 50–74, hypertension and diabetes prevalence (%).

3 CBS and MOH Health and social profile profile of localities, 2005–2009 [18], average monthly salary, 2009 National Insurance Institute data.

4 CBS and MOH Health and social profile profile of localities, 2005–2009 [18], based on Cancer Registry in Ministry of Health, average for 2005–2009 for all cancers, rate/100,000.

5 CBS Social survey, 2010, data for ages 20 and over

Among socio-economic factors, education had the largest number of significant correlations, showing an inverse relationship with SMRs for total mortality, total male and total female mortality, and for cancer and diabetes mortality. Unemployment rate showed a significant positive correlation with heart disease mortality, and rate of supplementary health insurance (a proxy measure of socio-economic status) had an inverse relation with cancer, diabetes and heart disease mortality. New immigrant proportion showed a significant negative correlation with chronic lower respiratory disease (CLRD) mortality. No significant correlations were found for average income (male and female).

Smoking and hypertension showed numerous significant positive correlations, with total mortality (total, male and female) and diabetes mortality, while hypertension also showed a significant association with cerebrovascular disease and cancer SMRs.

Cancer incidence was associated significantly with total and total male SMRs and also with kidney disease SMR, while diabetes prevalence was associated with the diabetes and cancer SMRs as well as total female mortality SMR.

## Discussion

We presented SMRs for the period of 2009–2013, which can be used to compare mortality in different regions with the national average, after controlling for differences in age and ethnic composition. We found statistically significant differences between geographical regions for total mortality and specific causes of death.

However, caution must be exercised in interpreting these differences. Langford has demonstrated [19] that where the number of expected cases is small, such as in small sub-districts, or diseases with few cases, relative risk, such as that shown by the SMR, is likely to have extreme values. Similarly, small differences in SMR are more likely to be statistically significant in areas with larger populations. We saw this in Fig. 1, where the confidence intervals were larger in smaller areas, and in Table 2, where differences in SMR values were larger for causes of death with small numbers, such as septicemia and influenza/pneumonia compared to cancer. This does not necessarily mean the differences are more meaningful. We therefore present results only for causes with large numbers of deaths, for a combined five year period, and concentrate on results with a high level of statistical significance, but still have to bear in mind that smaller areas and causes tend to have more extreme SMRs.

### Health policy implications of differences

We first note the relatively small range of SMR for total mortality, and that differences have narrowed compared to those reported by Ginsberg [3] for all the population in 1983–1986, also adjusted for age and ethnic origin, which ranged from 8% higher in the Ramla sub-district to 9% lower in the Jerusalem district. (Ginsberg did not report on Judea and Samaria due to the small number of deaths there in those years.) In addition, surprisingly our study did not show higher rates at the most significant level in any region for the leading cause of death in Israel [5], cancer. However, when we did not adjust for ethnicity, the variation in SMR’s was greater (Fig. 1 compared to Additional file 3).

These results show relative regional equality in Israel for total and cancer mortality, after “smoothing out” differences due to ethnicity, perhaps a compliment to equal regional health care coverage giving people living throughout the country access to mortality preventing healthcare, but also no doubt due to the small geographic size of Israel, with small distances between many regions allowing easy access to health facilities, and similar environmental influences. But the converse of this is that there remain differences in health associated with ethnicity, which affect mortality more significantly than regional factors. These mortality differences have been reported by the CBS and MOH [13, 14] and need to be addressed by health policy, as detailed below.

Similarly, in the amenable mortality study [8], also unadjusted for ethnicity, we found low rates in the Jerusalem and Central districts, but higher rates in the Northern district, which we did not find here, which probably reflect ethnic differences. In addition, since amenable mortality is defined as deaths from selected causes below age 75, it is possible that there is an excess of deaths at younger ages in the North from causes amenable to health care, compared to other regions. In Tel Aviv, we found the reverse, lower amenable mortality rates, but higher total SMR, perhaps reflecting older ages of mortality there.

We found the most significant socio-economic factor associated with mortality was years of education (Table 4), which was strongly inversely correlated with total mortality SMR, and the leading causes of cancer and diabetes. Filate et al. [15] similarly found significant inverse associations of cardiovascular disease rates with post-secondary education. In Israel, Jaffe et al. have shown a significant decrease in mortality with increasing education [20]. Hence, whatever can be done to increase the education level of all sectors of the population, should contribute to better health outcomes.

The most significant risk factor effect shown was with smoking, significantly correlated with SMRs for total and diabetes mortality. We recommend, as did Filate et al. who found similar results, that efforts to decrease smoking rates are an important health policy priority. Encouraging control of hypertension, also strongly associated with SMRs, particularly cancer, diabetes and cerebrovascular disease is also important.

We found few other significant correlations with socio-economic factors. Exceptions were unemployment, positively associated with heart disease mortality, as found by Filate et al., and supplementary health insurance inversely associated with diabetes, cancer and heart disease mortality. The absence of supplementary health insurance, an indicator of low socio-economic status in Israel where the rates of coverage are generally high (see Additional file 2), is seen to be associated with higher mortality from diabetes, cancer and heart disease.

Heart disease, the second leading cause of death, and diabetes showed more regional variations in SMR that were statistically significant than cancer. These diseases had significantly higher SMRs in the Northern and Haifa districts, and lower SMR’s in some Central and Southern sub-districts. Interestingly, this south to north gradient for heart disease was reported in the European atlas in other countries, too, such as the UK and Ireland [11], although its cause is unclear. Kidney disease also showed higher mortality than expected in the Haifa sub-district. The proportion of diabetics among new patients receiving renal replacement therapy in Israel has been rising [21], so this kidney disease mortality may be found together with the high diabetes and heart disease SMRs we found in this region, and perhaps reflect high prevalence of these diseases.

Israel reported higher rates of revascularization procedures in the Northern district in 2011, higher CABG rates in the Northern and Haifa districts, and higher PTCA rates in the Northern district, but also high revascularization rates in the Southern district [22]. It is to be hoped that early interventions with these procedures will prevent more severe heart disease and reduce its mortality in the north and in the Haifa sub-district, and that the lower mortality in the south will be maintained. These results may also support the importance of the MOH policy decision in recent years to increase cardiology facilities in the north.

Another important priority in preventing heart and kidney disease, diabetes and cancer, which would help reduce resulting mortality, is education to healthy living, particularly important in high mortality regions and for those of low socio-economic status. The inter-ministerial program for healthy and active living [23], for example, is an important initiative encouraging good eating habits and exercise.

Screening tests for cancer, mammography and guaiac faecal occult blood tests can contribute to reducing corresponding cancer mortality. In the USA, decreasing colorectal cancer incidence and mortality rates have been found with increasing screening, and Zauber discusses its connection with screening tests [24]. In Israel, too, where free, annual, high-sensitivity guaiac faecal occult blood tests was introduced in 2005, rates of incidence and mortality from colorectal cancer have been decreasing [25]. The program for Quality Indicators in Community Healthcare [26] has documented increases in coverage of these tests, but there is room for improvement, which should particularly be encouraged for occult blood tests in the Southern region where we found higher mortality than the national average.

### Septicemia

Septicemia is a severe condition defined as systemic inflammatory response syndrome which is often the immediate cause of death. However, unless nothing else at all is known about a patient, it should not be used as the underlying cause of death, as it is considered an ill-defined condition [27]. Rather, the source of the infection should also be written on the notification of death (NOD) form, if possible. Septicemia rates are very high in Israel compared to other countries, the 6th leading cause of death in 2010–2012 [5, 28], and this may be because the NOD form is not completed according to this directive. We have found hospital variations in proportion of deaths from septicemia, as well as whether it is listed as the only cause. These differences in NOD form completion may contribute to the regional significant differences in SMR for septicemia which we found.

In addition, to correct this problem, it is important to train physicians on how to complete the NOD form. The MOH is currently embarking upon a web based training program that might become compulsory for all medical graduates. We recommend speedy implementation of this program, for medical students and hospital physicians, particularly in those hospitals with high proportions of deaths from septicemia. In a recent MOH research initiative, hospital records are also being checked for some of these cases, to see if the correct underlying cause can be established.

Wang et al. [8] suggest that differences in sepsis mortality may reflect differences in sepsis treatment, or for example medical comorbidity, health behavior or socio-economic status.

The high mortality rate in general from septicemia, and in the Southern and Tel Aviv districts in particular, indicate the importance of encouraging prevention of acquired blood stream infections (BSI) in institutions. This is one of the aims of the MOH Center for Infection Control and Antibiotic Resistance which is responsible for directing and coordinating activities connected to infection control and prevention in medical institutions, and the issue of antibiotic resistance in institutions and in the community. The Center sets national policy, standards and interventional methods, and maintains data to be used as a basis for improving the quality and safety of treatment (https://www.health.gov.il/English/MinistryUnits/HealthDivision/InfectionControl/Pages/default.aspx). Their data from monitoring the acquired central line associated BSI rate in Israel have shown a 50% reduction in the infection rate in a 4 years period (unpublished). The Center has extended its ongoing interventions to a national program to prevent acquired healthcare infections which was begun in 2016. This includes staff training programs and incentives to encourage hospitals to invest resources in improving implementation of MOH guidelines and standards to prevent infections, and succeed in reducing acquired infection rates (https://www.health.gov.il/English/News_and_Events/Spokespersons_Messages/Pages/25012017_1.aspx).

Although this program should encourage hospitals to work on reducing infections, the available facilities and manpower also need to be improved in Israel. A recent article by Humphreys [29] reports studies showing that overcrowding and understaffing in hospitals can lead to increased acquired infections. This problem needs to be addressed in Israel, which has very high hospital occupancy rates and low population rates of practising nurses compared to most OECD countries [28]. Overcrowding may be particularly acute in the Southern district, where the age adjusted rate of hospital beds in 2011 was 1.6 per 100,000 population compared to the national average of 1.9, and 2.3 in the Jerusalem and Haifa districts. The new hospital in Ashdod may help alleviate this, but still more beds are needed nationwide. Also, hospital bed occupancy rates were particularly high in the Tel Aviv district, over 112% in 2011 compared to 98% in the Jerusalem district and 90–93% in other regions, and should be reduced [30].

### Accidents

Accidents had significantly higher SMRs in the Zefat and Be’er Sheva sub-districts. This was seen even more strongly in the SMRs for the largest group of accidents, motor vehicle traffic accidents, also significantly higher in the Judea and Samaria district and lower in Jerusalem. The high SMRs may reflect the longer distances traveled in these more sparsely populated peripheral areas, often on narrow, poorly maintained and badly lit roads, which increase the chance of accidents. In addition, when accidents do occur, it may take longer for emergency services to arrive. We recommend an improvement in the infrastructure of roads in these regions, and additional emergency health care centers which may help overcome this difference.

As Ginsburg notes [3], in the large urban areas of Jerusalem and Tel Aviv, there are lower average vehicle speeds. Since speed is a key risk factor in road traffic injuries [31], also shown in Israel in a study by Richter et al. [32], these lower speeds may lead to lower accident fatality rates and contribute to the lower SMRs for accidents we found in these regions.

### Explanatory factors

We found low SMRs for total mortality and for many causes, in the Jerusalem, Judea and Samaria and Central districts. In the Central district this may be accounted for by the high socio-economic level in many cities of its sub-districts Sharon, Petah Tiqwa and Rehovot,

In the Judea and Samaria district the socio-economic level is high only in a few settlements. But the population of the Judea and Samaria district is very young, with over 40% under 15, and only 3% over 65, compared to a national average of 28% and 10%, respectively (see Additional file 2). It is possible that age-standardization using 10 year age bands (for ages over 24) with an oldest age group of 75 and over, does not compensate adequately for such a different age composition and may help explain the very low SMRs found. In contrast to this, in sub-districts with older population, such as Tel Aviv and Haifa, where 14% and 16%, of the population are over 65, respectively, Ginsberg points out [3, 4] that the wide 75 and over age band tends to lead to an underestimation of expected deaths and consequently higher SMRs.

The SMR in the Jerusalem district, which has a relatively low socio-economic level, may also be influenced by the relatively large religious population there, and similarly in the Judea and Samaria district. A recent study by Sharoni et al. [33] showed that the social capital of the religious population, such as strong family and community connections and high level of volunteer activities may compensate for their lower socio-economic level, and explain their low mortality rates. Kark et al. [34] also found significantly lower mortality amongst the religious, in a study comparing religious and secular kibbutzim, as did Jaffe et al. [35] in a study on the effect of religiously affiliated neighborhoods on mortality. Hence, lower mortality among the religious population could contribute to the low SMRs in these regions. In particular, the SMR for suicide was significantly low in Jerusalem. The religious population there may be protected from suicide by religious prohibitions and spiritual beliefs, as by a strong cohesive community with shared values [36], although it may also be that suicide is under-reported due to religious stigmas, as noted in an Australian Parliamentary inquiry on suicide determination [37]. In the Tel Aviv district, a region with a less religious population, somewhat higher suicide mortality was found.

Surprisingly, although we found cancer incidence associated with total mortality, the association with cancer mortality was not significant. It is possible that this is due to high survival rates for some cancers [28], or mobility of the population after cancer diagnosis. The relatively high cancer survival rate may also explain the high association we found of cancer incidence with the SMR for kidney mortality, since kidney disease is a common complication of cancer [38] and cancer risk was found to be higher in patients with end-stage renal disease [39].

Despite significant variations in income levels and new immigrant proportions between sub-districts, the correlations of these factors with SMRs were not significant, unlike Filate et al. who found low income significantly associated with ischemic heart disease and immigrant population with cardiovascular disease. It appears that after controlling for differences in mortality due to age and ethnic group, income and immigration effects are small. It is also possible that because variations in income within sub-districts are great, the average income does not reflect the true income distribution, and in addition since Israel has had national health insurance since 1995 and 95% coverage before then, allowing universal access to medical treatment independent of income, the income effect may be low.

Another surprising finding for which we do not have an explanation, was the negative association of new immigrant proportion with CLRD mortality, particularly in view of recently reported *higher* respiratory mortality among immigrants from the Chernobyl area [40] .

### Changes over time

The mortality patterns we found were on the whole remarkably similar to those presented by Ginsberg [3]. Noteworthy differences include that the SMR for total mortality in the Ramla and Hadera sub-districts were no longer significantly high. In Ramla, this is probably due to the new socio-economically strong cities of Modi’in and Shoham.

### Addressing ethnic inequality

We have shown that ethnic factors appear to contribute more than regional factors to mortality inequalities, and need to be addressed. In a keynote presentation at a recent conference [41], Basharat highlighted the need to target the Arab population to improve their health outcomes, and reduce their high rates of obesity and diabetes, in particular by preventive medicine such as encouraging the use of whole-wheat breads by subsidies and education, and medical leadership through family physicians. An important program reported by the Clalit Health fund in the Sharon and Shomron area for the Arab population [42], included culturally attuned programs for healthy living amongst Arab women. Among successful outcomes was an increase in a quality index score for the region, becoming higher than the national average, increased drug usage for some diabetes drugs improving control, and increased flu vaccination coverage. Such programs must be encouraged and expanded.

Screening for cancer is another important preventive measure as discussed above, which can be encouraged for ethnic groups at higher risk for particular cancers.

### Strengths and limitations

The strength of our study is that it is based on complete mortality data over a period of five years, enabling regional comparisons at a leading cause level. Our standardization for age and ethnic group allows us to look for variations beyond differences in population composition.

However, our adjustment for ethnic group, based on country of birth, may not be adequate. As Ginsburg notes [3, 4], there may be considerable genetic and cultural differences between people born on the same continent, for example Yemen and Iran, who are grouped together for purposes of standardization, and similarly the Israeli born group is very heterogeneous, including offspring born to immigrants from all continents. Amongst the Arab population there are also different sub-groups.

Drawing conclusions from comparisons between different regions in Israel may be problematic, since to compare two population groups in a meaningful way, the variation between them should be larger than within them, as per Shaw et al. [13]. This requires relatively homogenous groups, of similar size, while the sub-districts in Israel vary greatly in size and many, particularly the larger ones, have a very heterogeneous population, with a wide range of socio-economic levels.

Our study is based on the underlying cause of death, which is coded by the CBS from notification of death forms completed by the physician who certifies the death. There may be regional differences in how physicians fill in the form, deciding on the chain of events leading to death and the underlying cause. For example, in our paper on high mortality rates from diabetes and renal failure [43], we discuss whether it is possible that these diseases are listed as the underlying cause instead of heart disease or stroke more often in Israel than other countries,. The choice and coding of underlying cause is done by a small number of coders at the CBS, and is unlikely to contribute to regional differences.

We attempted to look for associations using regional data, although our correlations are ecological in nature as we do not have individual level data on socio-economic and risk factors, and are therefore subject to potential ‘ecological fallacy’ (https://en.wikipedia.org/wiki/Ecological_fallacy). In addition, since many factors, such as education, diabetes and hypertension prevalence and unemployment, were survey based and subject to sometimes large relative sampling errors and the SMRs also had sometimes wide confidence limits, the results may be misleading. Therefore it is not surprising that correlations were not very high, although in general significant associations were as expected. In view of the multiple comparisons, we considered the Bonferroni correction. Only some of the total mortality and cancer SMRs with education and hypertension remaining statistically significant and maybe the above conclusions should be limited to them.

It would be useful if administrative data, such as that from the program for Quality Indicators in Community Healthcare [26] based on health fund records, were available by district and sub-district, which would enable research on regional differences with more accurate data.

## Conclusion

The SMR, adjusted for age, and ethnicity showed some districts that differ significantly from the national average, beyond that expected from differences in population structure.

Some of the regional differences may be attributed to differences in the completion of NOD forms by physicians. This needs to be addressed by efforts to improve reporting of causes of death, by training of medical students and refresher courses for qualified physicians.

The relatively small significant differences found after our adjustment, shows the importance of targeting factors causing ethnic inequalities, rather than regional ones. Recommendations include raising the education level, reducing smoking, control of hypertension, encouraging healthy lifestyles and screening for cancer. This is particularly important for those of a low socio-economic level.

## Additional files

Additional file 1: Map of Israel showing districts and sub-districts. (DOCX 303 kb)

Additional file 2: Demographic, socio-economic and health characteristics of sub-districts of Israel. (XLSX 22 kb)

Additional file 3: SMR by sub-district, 2009–2013, standardized for age only, with 95% CI error bars. (DOCX 163 kb)

Additional file 4: SMR for total mortality by district, sub-district and gender, 2009–2013, with 95% confidence intervals (Table 1 with 95% CI). (XLSX 11 kb)

Additional file 5: SMR for mortality by leading causes of death, district and sub-district, 2009–2013, with 95% confidence intervals (Table 2 with 95% CI). (XLSX 14 kb)

Additional file 6: SMR for mortality by selected causes of death, by district and sub-district, 2009–2013, with 95% confidence intervals (Table 3 with 95% CI). (XLSX 15 kb)

## Abbreviations

CBS: Central Bureau of Statistics
CLRD: Chronic Lower Respiratory Disease
MOH: Ministry of Health
NOD: Notification of death
SMR: Standard Mortality Ratio

## References

[1] Health in Israel: selected data. Ministry of Health, 2010 (Hebrew and English) http://www.health.gov.il/PublicationsFiles/HealthIsrael2010.pdf.

[2] Ginsberg GM (1983). Standardized mortality ratios for Israel, 1969–78. Isr J Med Sci.

[3] Ginsberg GM (1992). Standardized mortality ratios for Israel, 1983–86. Isr J Med Sci.

[4] Ginsberg GM, Tulchinsky TH, Salahov E, Clayman M (2003). Standardized mortality ratios by region of residence, Israel, 1987–1994: a tool for public health policy. Public Health Rev.

[5] Goldberger N, Aburbeh M, Haklai Z. Leading causes of death in Israel, 2000–2012″ Ministry of Health. 2015 (Hebrew and English publications). https://www.health.gov.il/PublicationsFiles/Leading_Causes_2012E.pdf.

[6] Central Bureau of Statistics, Israel: Adjusted mortality rates, by cause, district and sub-district, ages 45+, average 2005–2009. http://www.cbs.gov.il/briut/new/t2005_2009.xls.

[7] Central Bureau of Statistics, Israel: Adjusted mortality rates, by cause, district and by population group, average 2006–2008. http://www.cbs.gov.il/briut/new/t2006_2008.xls.

[8] Goldberger N, Haklai Z (2012). Mortality rates in Israel from causes amenable to health care, regional and international comparison. Israel J of Health Policy Research.

[9] Mortality atlas of Canada Canada department of National Health and Welfare, Statistics Canada 1980.

[10] Pickle LW, Mungiole M, Jones GK, White A (1996). Atlas of United States mortality.

[11] Eurostat: Health statistics – Atlas on mortality in the European Union, 2002 and 2009.

[12] Trewn D. Mortality Atlas, Australia 1997–2000. Australian Bureau of Statistics. 2002;

[13] Shaw M, Orford S, Brimblecombe N, Dorling D (2000). Widening inequality in mortality between 160 regions of 15 European countries in the early 1990s. Soc Sci Med.

[14] Muller-Nordhorn J, Binting S, Roll S, Willich SN (2008). An update on regional variation in cardiovascular mortality within Europe. Eur Heart J.

[15] Filate WA, Johansen HL, Kennedy CC, Tu JV (2003). Regional variations in cardiovascular mortality in Canada. Can J Cardiol.

[16] Wang HE, Devereaux RS, Yealy DM, Safford MM, Howard G (2010). National variation in United States sepsis mortality: a descriptive study. Int J Health Geogr.

[17] Ulm K (1990). A simple method to calculate the confidence interval of a standardized mortality ratio (SMR). Am J Epidemiol.

[18] Central Bureau of Statistics, Israel: Health and social profile of localities in Israel, 2005–2009. http://www.cbs.gov.il/webpub/pub/text_page.html?publ=105&CYear=2009&CMonth=12.

[19] Langford IH (1994). Using empirical Bayes estimates in the geographical analysis of disease risk. Area.

[20] Jaffe DH, Neumark YD, Eisenbach Z, Manor O (2008). Educational inequalities in mortality among Israeli Jews: changes over time in a dynamic population. Health Place.

[21] Calderon-Margalit R, Gordon ES, Hoshen M, Kark JD, Rotem A, Haklai Z. Dialysis in Israel, 1989–2005 - time trends and international comparisons. Nephrol Dial Transplant. 2008(23):659–64. 10.1093/ndt/gfm597 17881428

[22] OECD Health policy studies: Geographic variations in health care: What do we know and what can be done to improve health system performance? 2014 OECD Publishing 10.1787/9789264216594-en.

[23] “It is possible to be healthy”- The national program for active and healthy living http://cms.education.gov.il/EducationCMS/Units/Mazkirut_Pedagogit/Briut/TochniyoBriut/EfshariBari/odot.htm. (Hebrew).

[24] Zauber G (2015). The impact of screening on colorectal cancer mortality and incidence – has it really made a difference?. Dig Dis Sci.

[25] Silverman B, Keinan-Boker L, Lifshitz I, Fishler Y, Dichtiar R: Colorectal cancer in Israel – update. Ministry of Health 2014: http://www.health.gov.il/PublicationsFiles/ICR_21072014.pdf (Hebrew).

[26] Manor O, Shmueli A, Ben-Yehuda A, Paltiel O, Calderon R, Jaffe DH (2014). National Program for quality indicators in community healthcare in Israel, report for 2011–2013.

[27] Health Information Systems Knowledge Hub: Handbook for doctors on cause-of-death certification, 2012. http://www.getinthepicture.org/sites/default/files/resources/Handbook%20for%20doctors%20on%20cause-of-death%20certification.pdf.

[28] OECD statistical data, http://stats.oecd.org/#.

[29] Humphreys H (2006). Overcrowding, understaffing and infection in hospitals. Ir Med J.

[30] Ministry of Health: Inpatient institutions and day care units in Israel, 2011. https://www.health.gov.il/UnitsOffice/HD/MTI/info/Pages/Inpatient_Institutions.aspx. (Hebrew).

[31] WHO Facts Road safety – Speed http://www.who.int/violence_injury_prevention/publications/road_traffic/world_report/speed_en.pdf.

[32] Richter E (1981). Death and injury from motor vehicle crashes in Israel; epidemiology, prevention and control. Int J Epidemiol.

[33] Sharoni C, Tchernichovsky D: The secret of the connection between Orthodoxy and Health. NIHP 2015. 11^th^ Annual conference on Health Policy.

[34] Kark JD, Shemi G, Friedlander Y, Martin O, Manor O, Blondheim SH (1996). Does religious observance promote health? Mortality in secular vs religious kibbutzim in Israel. Am J Public Health.

[35] Jaffe DH, Eisenbach Z, Neumark YD, Manor O (2005). Does living in a religiously affiliated neighborhood lower mortality?. Ann Epidemiol.

[36] Van Praag H. The role of religion in suicide prevention. In: Wasserman D, Wasserman C, editors. Oxford textbook of suicidology and suicide prevention: a global perspective. Oxford University Press; 2009:7–12.

[37] Senate Community Affairs Reference Committee: The Hidden Toll: Suicide in Australia. 2010. http://www.aph.gov.au/Parliamentary_Business/Committees/Senate/Community_Affairs/Completed_inquiries/2008-10/suicide/report/index

[38] Humphreys B, Soiffer RJ, Magee CC: Renal failure associated with cancer and Its treatment: An update J Am Soc Nephrol 2005;16: 151–161,. doi: 10.1681/ASN.2004100843 10.1681/ASN.2004100843 15574506

[39] Butler AM, Olshan AF, Kshirsagar AV, Edwards JK, Nielsen ME, Wheeler SB, Brookhart MA (2015). Cancer incidence among US Medicare ESRD patients receiving hemodialysis, 1996–2009. Am J Kidney Dis.

[40] Slusky DA, Cwikel J, Quastel MR (2017). Chronic diseases and mortality among immigrants to Israel from areas contaminated by the Chernobyl disaster: a follow-up study. Int J Public Health.

[41] Basharat B:: The Israeli health system as reflected in the Arab population. NIHP 2014. 10^h^ Annual conference on Health Policy.

[42] Sadeh Z, Zimmerman P, Ron A, Segev D, Gidoni Y, Nimni K: Towards health promotion in the Arab sector in the Sharon-Shomron region. The society for quality of health in medicine, 21^st^ conference, 2014.

[43] Goldberger N, Applbaum Y, Meron J, Haklai Z (2015). High Israeli mortality rates from diabetes and renal failure – Can international comparison of multiple causes of death reflect differences in choice of underlying cause?. Israel J of Health Policy Research.

[44] Atlas of mortality in Israel, 2009–2013. Ministry of Health, 2016 (Hebrew) http://www.health.gov.il/PublicationsFiles/DeathAT2009_2013.pdf

