# Supplementary material for: Regional variations in mortality and causes of death in Israel, 2009–2013
Source: Isr J Health Policy Res. 2017 Aug 1;6:39. doi: 10.1186/s13584-017-0164-1 (PMC5537988; doi:10.1186/s13584-017-0164-1)
Supplement: Supplementary file 3 — SMR by sub-district, 2009–2013, standardized for age only, with 95% CI error bars. (DOCX 163 kb) [file 13584_2017_164_MOESM3_ESM.docx]

SMR by sub-district, 2009-2013**,** standardized for age only, with values and 95% CI error bars
