# Supplementary material for: Regional variations in mortality and causes of death in Israel, 2009–2013
Source: Isr J Health Policy Res. 2017 Aug 1;6:39. doi: 10.1186/s13584-017-0164-1 (PMC5537988; doi:10.1186/s13584-017-0164-1)
Supplement: Supplementary file 3 [file 13584_2017_164_MOESM1_ESM.docx]

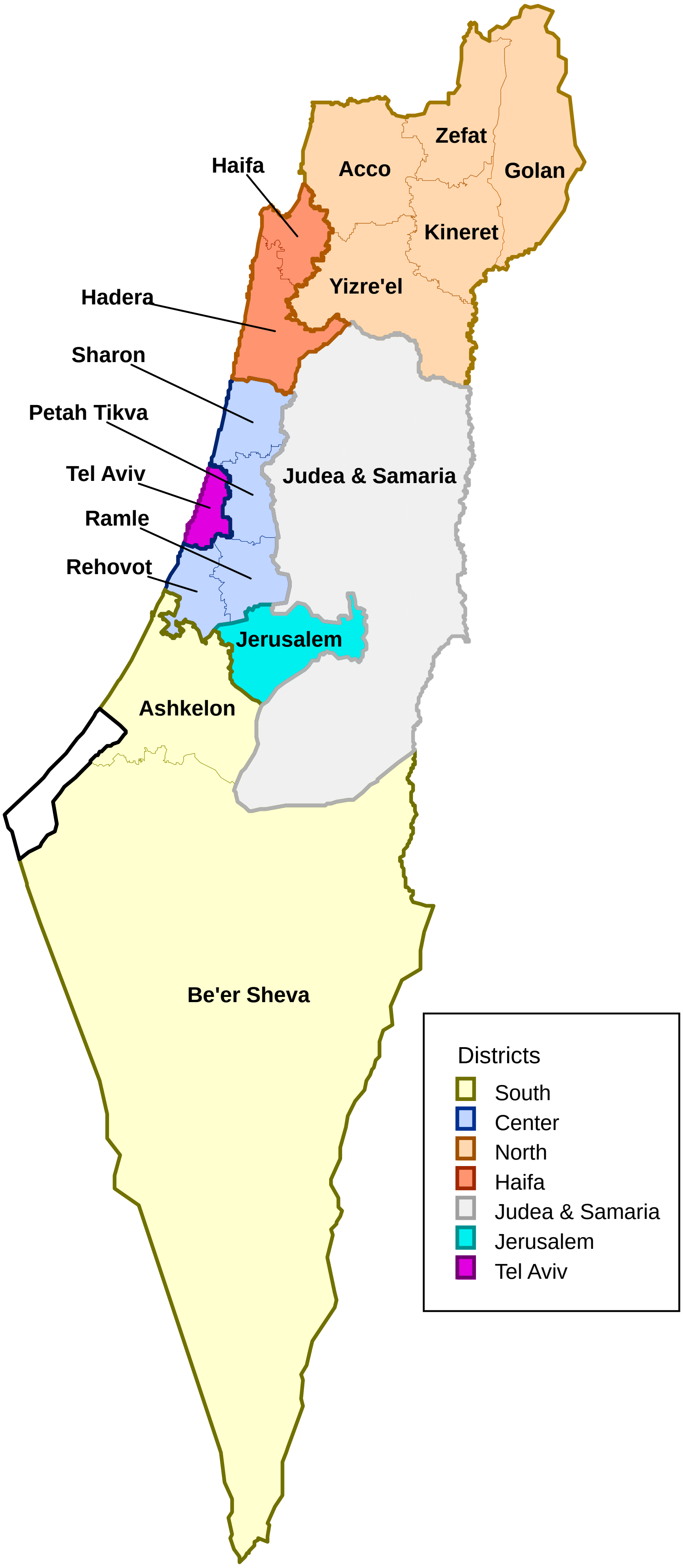
Haifa
Acco
Zefat
Golan
Kineret
Yizre'el
Hadera
Sharon
Petah Tikva
Tel Aviv
Ramle
Rehovot
Judea & Samaria
Jerusalem
Ashkelon
Be'er Sheva
Districts
South
Center
North
Haifa
Judea & Samaria
Jerusalem
Tel Aviv
